# Protocol for *Get Moving*: a randomised controlled trial to assess the effectiveness of three minimal contact interventions to promote fitness and physical activity in working adults

**DOI:** 10.1186/s12889-015-1654-0

**Published:** 2015-03-27

**Authors:** Andrew JM Cooper, Katie Dearnley, Kate M Williams, Stephen J Sharp, Esther MF van Sluijs, Soren Brage, Stephen Sutton, Simon J Griffin

**Affiliations:** MRC Epidemiology Unit, University of Cambridge, School of Clinical Medicine, Institute of Metabolic Science, Cambridge Biomedical Campus, Cambridge, UK; Behavioural Science Group, Primary Care Unit, Department of Public Health and Primary Care, University of Cambridge, Institute of Public Health, Robinson Way, Cambridge, CB2 0SR UK; Primary Care Unit, Department of Public Health and Primary Care, University of Cambridge, Institute of Public Health, Robinson Way, Cambridge, CB2 0SR UK

**Keywords:** Physical activity, Intervention, Internet, Monitoring and feedback, Self-monitoring

## Abstract

**Background:**

Web-based interventions for physical activity offer several advantages over face-to-face, print-and telephone-based interventions and are scalable and potentially cost-effective. Recent reviews of web-based interventions in adults show that they have positive but small effects on physical activity but identify a number of limitations including a reliance on self-report measures of outcome. This trial used an objective measure of physical activity to assess the effectiveness of three minimal contact interventions: 1) A multi-component web-based intervention incorporating objective monitoring and graphical feedback of physical activity; 2) A version of the first intervention that consisted only of objective monitoring plus web-based graphical feedback; and 3) Self-monitoring of physical activity using a paper diary.

**Methods/design:**

*Get Moving* is an individually randomised controlled trial with allocation of 488 participants to one of three interventions or to a no-intervention control group. Participants are physically inactive working adults aged 18–65 years. They attended a baseline assessment session at which anthropometric, biological and questionnaire measures were taken and they completed a treadmill exercise test. They then wore a combined movement and heart rate monitor for six days and nights before being randomised to one of the four trial arms. The baseline measures were repeated at the follow-up assessment which took place approximately 12 weeks post-randomisation, conducted by staff blind to group allocation. Participants wore the movement and heart rate monitor for six days and nights before this. The co-primary outcomes are: physical activity energy expenditure measured using individually calibrated combined heart-rate and movement data; and cardiorespiratory fitness measured using a sub-maximal treadmill exercise test.

**Discussion:**

Strengths of the trial include the use of an objective measure of physical activity, a measure of cardiorespiratory fitness, relatively large sample size and the use of robust methods of randomisation, allocation concealment and blinding to outcome assessment. *Get Moving* will contribute to the evidence base on minimal contact interventions for increasing physical activity. The interventions could be implemented in other settings such as primary care.

**Trial registration:**

ISRCTN31844443. Registered 18 June 2010.

## Background

Regular physical activity plays a pivotal role in the prevention and treatment of numerous health related conditions including obesity, depression, anxiety, hypertension, type 2 diabetes, cardiovascular disease, osteoporosis, stroke and breast and colon cancers [1]. Physical inactivity is now recognised as the fourth leading risk factor for global mortality [2], accounting for 6% of all deaths. As such, the World Health Organization (WHO) recommends that adults should do at least 150 minutes (2.5 hours) of moderate-intensity physical activity each week [2]. Furthermore, studies examining the dose–response relationship of physical activity have shown that higher intensity activity (i.e. vigorous-intensity physical activity) improves cardiorespiratory fitness and provides health benefits which are comparable with, and in some cases even greater than, those observed for moderate-intensity activities [3].

Despite the health benefits of physical activity however, insufficient physical activity remains highly prevalent. In the UK more than half of all adults report not meeting recommended levels of physical activity – which is likely to be an optimistic figure given that 95% are not meeting recommended levels when activity is measured objectively [4]. The cost of inactivity to the UK National Health Service (NHS) was estimated at £1.06 billion in 2002, not accounting for the indirect costs resulting from days lost due to sickness absence, premature mortality, private healthcare costs and home care [5]. International reviews conclude that reversal of this physical inactivity pandemic requires public health programmes to encourage increasing activity at societal level, but also interventions to help high risk individuals to increase their levels of physical activity [6].

Previous randomised controlled trials have demonstrated the effectiveness of physical activity promotion among high-risk individuals for reducing the risk of several major non-communicable diseases such as type 2 diabetes [7]. However, these trials have typically been face-to-face interventions – that is, interventions delivered in person – and have been overseen by primary care doctors, physiotherapists, health visitors, health educators, specialist nurses and physical trainers, thereby making them expensive and difficult to implement in real-world settings. Furthermore, greater population health gain will be achieved through shifts in the overall distribution of physical activity as opposed to targeting high-risk groups. The major challenge at this point therefore lies in creating and delivering physical activity interventions which are effective, scalable and affordable to the general adult population. Programmes that deliver an intervention without face-to-face contact may be one way of overcoming these challenges.

In recent years, there has been increasing interest in developing and evaluating web-based interventions for physical activity [8]. These offer several advantages over face-to-face interventions and those based on printed materials and telephone contact, including the potential to reach a large number of people at a relatively low cost, the ability to provide 24-hour access to intervention materials, and the capacity to provide immediate and tailored feedback. Recent reviews of web-based interventions in adults have shown that they have positive but small effects on physical activity. However, they identify a number of methodological limitations of published trials including a reliance on self-report measures of physical activity [9,10].

In the Get Moving trial (*Get Moving*), we are evaluating the effectiveness of a multicomponent web-based intervention developed by Imperative Health (part of AXA ICAS) and designed to support behaviour change in the domains of physical activity and diet. A key feature of the intervention is objective self-monitoring of physical activity. Participants are issued with a tri-axial wrist-worn accelerometer (activity band) from which physical activity data can be wirelessly uploaded to the website where they can view graphs of their progress. Objective monitoring and feedback may facilitate behaviour change in two ways. First, many physically inactive people are not aware of being inactive [11], so feedback may help to motivate them to change. Second, continuous monitoring and feedback enables people to track their progress towards a goal, which is consistent with self-regulation theories of behaviour change such as control theory [12].

Pedometers are commonly used in monitoring and feedback interventions, and there is evidence for the effectiveness of pedometer-based interventions [13,14]. However, few studies to date have evaluated interventions that combine objective monitoring of physical activity with web-based feedback. Richardson and colleagues found a significant increase in physical activity from before to after a six-week intervention in which participants (people with type 2 diabetes) used pedometers with USB ports, uploaded step-count data to a website and received automated feedback [15]. However, this intervention has not been evaluated in a randomized controlled trial. Slootmaker et al. [16] conducted a trial of the PAM (personal activity monitor) system in 102 young office workers. The PAM is a uni-axial accelerometer typically worn on the waist and continuously displays a score showing the cumulative amount of physical activity performed in that day. The information could be uploaded to a website which provided tailored advice and graphically displayed the participant’s progress. The intervention had no significant effect on physical activity or aerobic fitness at three- and eight-month follow-up compared with an information leaflet containing general physical activity recommendations. However, a similar trial in secondary school children found significant effects of the intervention [17]. Physical activity was measured by self-report in both trials.

In the *Get Moving* Trial, in addition to the full multi-component web-based intervention incorporating objective monitoring and graphical feedback of wrist-based physical activity monitoring with an online coaching engine (individualised in response to objective activity data), we include a version of the intervention which consists only of objective monitoring plus web-based graphical feedback. This enables us to test whether objective monitoring and feedback on its own is an effective intervention for physical activity and whether the additional components in the full intervention including the coaching element have any added benefit. The third and final intervention will be self-monitoring of physical activity using a paper-based diary. This technique has been used in numerous physical activity interventions but few studies have attempted to test it in isolation. The review by Fair [18] identified three such trials [19-21] which yielded a non-significant average effect. Self-monitoring using a diary might be expected to facilitate behaviour change through some of the same mechanisms as the objective device-based monitoring with web-based feedback. However, there are potentially important differences between the two approaches that may influence their relative efficacy. For example, the diary method will provide less complete and less quantitative information and therefore cruder feedback about physical activity compared with objective monitoring; the feedback differs in format (diary entries versus activity graph displayed on a webpage); and the monitoring task (writing in the diary versus uploading data from a wearable device) is carried out daily, which may affect engagement with and adherence to the intervention.

### Objectives

The main objective of *Get Moving* is to assess the effectiveness of three minimal contact interventions on objectively measured physical activity energy expenditure (PAEE; a measure of total physical activity) and cardiorespiratory fitness in a general adult population. The interventions will be compared with each other and with a no-intervention control group.

## Methods/Design

### Study design

**Figure 1:**
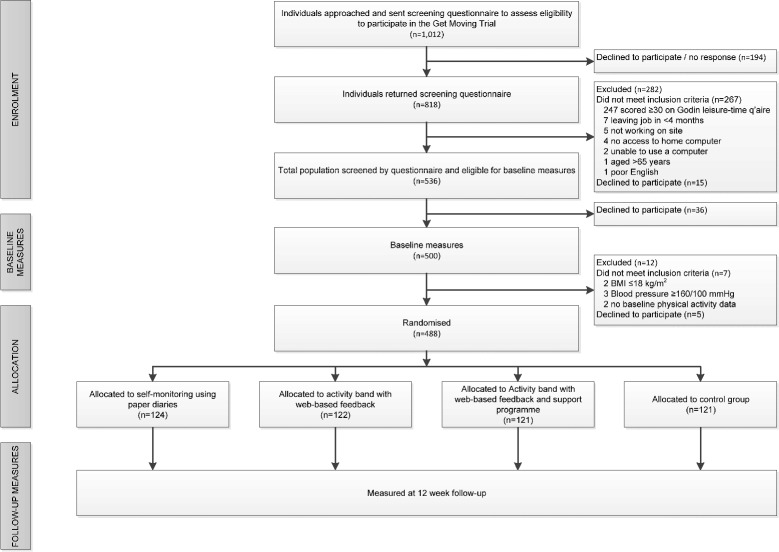
**Flow of participants through the** ***Get Moving*** **Trial.**

*Get Moving* is a parallel group, open label, randomised controlled trial with allocation of 488 participants to either no intervention (control group) or to one of three intervention groups: self-monitoring using paper-based diaries (diary group), activity band with web-based feedback (activity band group), or activity band with web-based feedback plus online coaching engine (activity band plus group). The co-primary outcomes are: (1) PAEE measured using individually calibrated combined heart-rate and movement sensing [22,23], and (2) cardiorespiratory fitness measured using heart rate response to a sub-maximal treadmill exercise test. The secondary outcomes are: anthropometric measures (body mass index (BMI), body fat %, weight and waist circumference), blood pressure, plasma vitamin C levels, biochemical measures (HbA_1c_, fructosamine, cholesterol and triglycerides), Short Form 8 (SF-8) health survey questionnaire, theory of planned behaviour (TPB) measures, perceived stress, self-monitoring behaviour and self-reported recent physical activity (RPAQ). Ethical approval was obtained from Cambridge Central NHS Research Ethics Committee (Ref 09/H0308/3). The design of the trial and flow of participants are shown in Figure 1.

### Recruitment

Individuals working or studying on the Cambridge Biomedical Campus (including Addenbrooke’s Hospital, Cambridge, UK) aged 18–65 years were eligible to take part in the study. Contact was made with each institution on the Biomedical Campus to establish an appropriate contact, who was asked to circulate a generic advert to staff and students, via newsletter, email, posters and/or via their institution’s intranet, with the aim of reaching all staff and students on the Campus. Recruitment stands were run in areas frequented by staff and students, e.g. staff canteen and lobby areas. Individuals who registered an interest in taking part in the study were sent an information sheet about the study and a brief questionnaire to complete and return to assess the first of a three stage eligibility assessment process (excluded if physically active (scoring ≥30 on the Godin Leisure-Time Exercise Questionnaire (GLTEQ) for moderate-to-vigorous activities [24]); were instructed by their GP not to engage in regular physical activity; were unable to walk briskly on the flat for 15 minutes without help). If an individual met this stage of eligibility criteria, they were contacted by telephone to go through the second stage of eligibility screening (excluded if they were participating in another clinical randomised trial; taking ≥100 mg/day of Atenolol or an equivalent amount of another beta-blocker; were pregnant; planning on leaving their position on the Biomedical Campus within 4 months of recruitment; did not have access to or were unable to operate an internet-connected home PC running the minimum specification requirements set by Imperative; or could not use an English-language website). Potential participants who remained eligible discussed participation in the trial in more detail with the study team by telephone and were given the opportunity to ask questions prior to booking an appointment to attend for baseline measures.

#### Baseline assessment

On arriving at the measurement centre, having fasted for the preceding one hour (including abstaining from nicotine, caffeine and vigorous exercise), individuals provided fully informed written consent following a discussion about the study with the measurement team. Height, weight and blood pressure were measured first to check the third stage exclusion criteria (individuals whose BMI was ≤18 kg/m^2^, or who had a mean blood pressure ≥160/100 mmHg – those routinely excluded by the Imperative Health system as a safety precaution). This was explained to potential participants prior to their attendance at the measurement visit in the information sheet and also during the telephone call. Individuals who met all inclusion criteria continued with the visit. All baseline information was collected prior to randomisation. Participants had anthropometric and biological measurements taken (see Measures section and Table 1 for further details). Participants then completed a treadmill exercise test which was terminated when 80% of their age-predicted maximum heart rate (i.e. 208 minus 0.7*age) was reached. Predicted maximal cardio-respiratory fitness (VO_2max.pred_) was estimated using extrapolation of the heart-rate to VO_2_ relationship to age-predicted maximal heart rate. Participants were asked to provide demographic information and to complete a number of questionnaire measures (see Measures section and Table 1). Anthropometric and clinical measurement values were concealed from participants unless they specifically asked to see the results, thereby minimising the possibility of behaviour change associated with clinical feedback and to maximise participant retention at follow-up. Before leaving the measurement centre, participants were fitted with a combined movement and heart rate monitor (Actiheart, CamNtech, Cambridge, UK) and were instructed to wear the monitor for six days and nights continuously. They were instructed on how to change ECG electrodes (supplied), both verbally and in writing with depiction of correct placement. In the event that an insufficient amount of valid data was collected from the combined monitor following two separate wear periods, the participant was not able to continue in the study.

**Table 1:** **Measures used in the Get Moving Trial**

**Measure(s)**	**Screening**	**Baseline visit**	**12-week follow-up visit**	**Post 12-week follow-up**
**Co-primary outcome measures**				
Physical activity		*√*	*√*	
Cardiorespiratory fitness		*√*	*√*	
**Anthropometric measures**				
Height		*√*		
Weight		*√*	*√*	
Waist circumference		*√*	*√*	
Body fat %		*√*	*√*	
**Biological measures**				
Blood pressure, heart rate, ECG (done at follow-up for participants that need a medical review), HbA_1c_, fructosamine, total cholesterol, HDL- and LDL-cholesterol, triglycerides, plasma vitamin C		*√*	*√*	
**Degree of intervention use**				
Paper-based physical activity diary use (Diary group)				*√*
Use of physical activity band from Imperative Health (Activity Band & Activity Band Plus groups)				*√*
No. of times the Imperative Health website was visited (Activity Band & Activity Band Plus groups)				*√*
**Demographic measures**				
Date of birth	*√*	*√*		
Sex	*√*	*√*		
Living situation		*√*		
Number of children in household		*√*		
Highest education level		*√*		
Ethnic origin		*√*		
**Psychological measures**				
Theory of planned behaviour questionnaire		*√*		
Theory of planned behaviour monitoring questionnaire (post-randomisation questionnaire)	Completed two weeks post randomisation			
Conscientiousness questionnaire		*√*		
**Self-reported health behaviours**				
Recent physical activity (R-PAQ)			*√*	
Functional status (SF-8)		*√*	*√*	
Perceived stress (4-item PSS)		*√*	*√*	
Self-report habit index questionnaire		*√*	*√*	
Gym membership		*√*	*√*	
Smoking status		*√*	*√*	
Vitamin supplement use		*√*	*√*	
Prescribed medications		*√*	*√*	
Frequency of self-weighing		*√*	*√*	
**Other questionnaires/single-item questions**				
Godin Leisure-Time Exercise Questionnaire (GLTEQ)	*√*			
Participating in other research	*√*			
GP advised against doing physical activity	*√*			
Beta-blocker use	*√*			
Pregnancy status	*√*			
Able to walk briskly for ≥15 minutes	*√*			
Access to and ability to use a home PC and internet	*√*			
Ability to use an English language website	*√*			
Leaving job in next 16 weeks	*√*			
Self-report of doctor diagnosed diabetes		*√*	*√*	
Rose Angina questionnaire		*√*	*√*	
Main study goal		*√*		
Job satisfaction			*√*	
Contact with other study participants (yes/no)			*√*	
Self-monitoring behaviour			*√*	
Use of physical activity advice websites (yes/no)			*√*	
Satisfaction with study participation				*√*
Programme evaluation questionnaire				*√*
Absence from work due to illness			*√*	

#### Randomisation, allocation concealment and blinding

Once the combined monitor had been returned and the recorded data checked for quality and quantity (≥35 hours), participants were randomly allocated to one of the four study groups (Control, Diary, Activity Band or Activity Band Plus). Randomisation lists were prepared using Stata [25] within strata defined by age (<45, ≥45 years), sex and BMI (<27, ≥27 kg/m^2^) using a block size of 8. Participants in the ‘Control’ and ‘Diary’ groups were sent their randomisation allocation by internal post. Participants in the ‘Activity Band’ and ‘Activity Band Plus’ groups were told their allocation at their place of work and were given the necessary equipment and instructions on how to proceed during the 12 week intervention. The measurement team were blinded to the participants’ group allocation throughout the trial.

#### Interventions

##### Control group

Participants randomised to this group received no intervention.

##### Diary

Participants were given a pocket-sized, paper-based physical activity diary and advised to record, on a daily basis, each time they engaged in physical activity for the duration of 10 minutes or more. Participants were instructed to record whether they undertook any activity (yes/no), the type of activity undertaken, the time they began the activity and its total duration, for the entire 12 week intervention period.

##### Activity band with web-based feedback (Activity Band)

**Figure 2:**
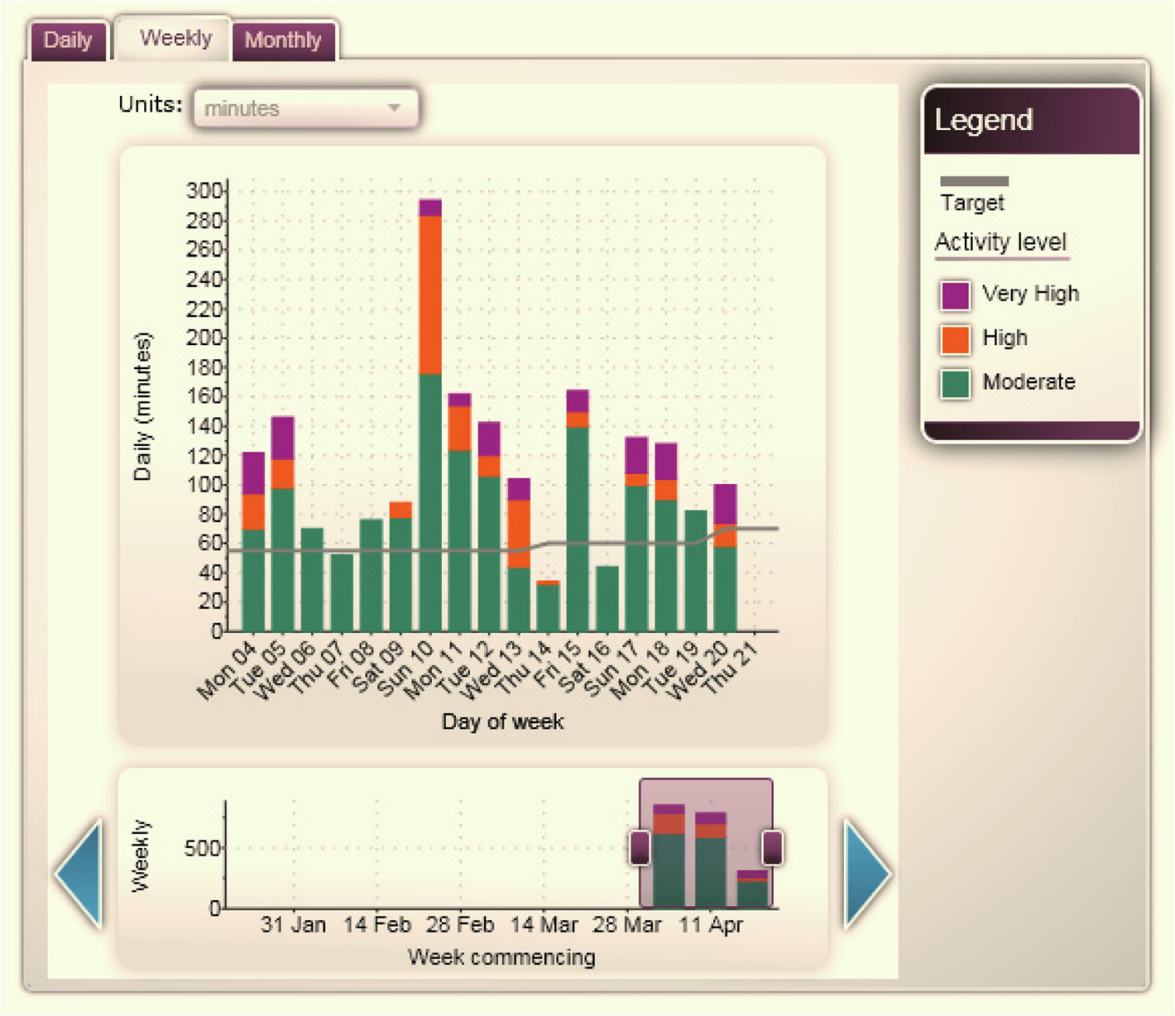
**Example of an Activity graph.**

Participants in this group were issued with a physical activity accelerometer (Activity Band, Imperative Health, AXA ICAS Limited, East Sussex, UK) and were asked to start using the band to monitor their physical activity. The Activity Band is a wrist-worn device containing a tri-axial accelerometer similar to the one described by Esliger et al. [26]. It is able to store movement data for up to two weeks until it is uploaded via Bluetooth to the Imperative Health website, which displays the physical activity data in graphical format. Participants were instructed to activate their account within one week of receiving the intervention and to log on to their account to upload their movement data and view their activity graphs at least once per week. The activity graphs numerically and graphically show the number of minutes of physical activity undertaken each day, each week and each month, separated into three different activity intensities (moderate, high and very high intensity activity). If no account activity was detected for a continuous period of 15 days, Imperative Health liaised with the study team who contacted the participant to find out if they were experiencing any technical problems with the programme or were no longer interested in receiving the intervention. (Figure 2).

##### Activity band with web-based feedback plus online coaching engine (Activity Band Plus)

Participants in this group were provided with the same physical activity band as the ‘Activity Band’ group (see above). In addition, participants were provided with a Bluetooth enabled weighing scale to enable self-monitoring of body weight; access to the nutritional component of the program for monitoring calorie intake, and access to the full Imperative Health online coaching engine (http://www.imperativehealth.com). The coaching engine is a web-based automated dialogue system which serves as a “virtual coach” to help individuals change their health-related behaviours. Earlier versions of Imperative have been tested in previous studies [27,28]. This system provides a more interactive experience for participants compared with the ‘Diary’ only and the ‘Activity Band’ intervention groups. As with the ‘Activity Band’ group, participants were instructed to activate their account within one week of receiving the intervention and to log on to their account to upload their movement data and view their activity graphs at least once per week. The system uses the activity data from the physical activity band and the weight data from the Bluetooth-enabled weighing scales to provide numerical and graphical feedback to the user regarding their progress. The system also contains structured daily meal plans, a food diary for recording all food and drink consumed, a physical activity planner to help with planning structured exercise, progress graphs with physical activity levels and weight loss goals displayed in graphical format, and a tailored message service which provides reminders for exercise and feedback to encourage the setting and meeting of goals. Participants were advised to use as many of these elements as they desired, but full engagement was encouraged. As with the ‘Activity Band’ group, if no account activity was detected for a continuous period of 15 days then Imperative Health liaised with the study team, who contacted the participant to find out if they were experiencing any technical problems with the programme or were no longer interested in receiving the intervention.

#### Post-randomisation questionnaire

All participants, irrespective of study group allocation, were sent a questionnaire by email two weeks after the date of randomisation to assess the determinants of monitoring behaviour and were asked to complete and return it to the study team. See Measures section for details of content.

#### Follow-up assessment

Approximately eight weeks post-randomisation, participants were contacted by telephone by a member of the study team to arrange a convenient date and time to attend for a follow-up visit approximately 12 weeks post-randomisation. At week 10 participants were asked to wear the combined movement and heart rate monitor for six days and nights continuously and to return it to the study co-ordination office along with any intervention equipment prior to their 12-week visit (to ensure the measurement team remained blinded to the intervention allocation). Participants were asked not to mention their randomised group to the measurement team at the follow-up visit. In the event that an insufficient amount of valid data was collected from the combined monitor, the participant was provided with another monitor at their follow-up visit to wear for the following six days and nights.

As for the baseline visit, all participants fasted for one hour (including abstaining from nicotine, caffeine, and vigorous exercise), had anthropometric and biological measurements taken, completed a sub-maximal treadmill test and were asked to complete questionnaire measures (see Table 1).

If, after repeated efforts to book a convenient appointment, a participant was unable to attend for their follow-up measurement visit, a postal alternative was offered which included questionnaires and the combined monitor for self-attachment. Participants who needed a repeat blood test (samples lost or degraded or insufficient blood volume taken) were offered the opportunity to return for a repeat blood test or to have the test repeated at their GP surgery. Participants agreeing to complete postal measures were followed up for a maximum of six months, after which time the participant was classified as ‘lost to follow-up’.

#### Programme evaluation questionnaire

A randomised group-specific evaluation questionnaire was sent to all participants by email after attending their follow-up visit (again to maintain blinding of the measurement team taking follow-up measures). See Measures section below for details of content.

#### Health report

After receipt of the evaluation questionnaire, all participants were sent a health report containing their physiological and clinical measurements (including measures of fitness and physical activity, blood pressure, weight, BMI, body fat percentage, waist circumference, blood glucose and total cholesterol levels) from both their baseline and 12 week visits. A copy of this report was also sent to their GP for information, together with the clinical results from both visits.

#### Measures

Table 1 shows the measures taken at each stage.

##### Cardiorespiratory fitness and free-living physical activity (co-primary outcome measures)

Cardiorespiratory fitness was assessed at baseline and again at 12 weeks using a ramped treadmill protocol consisting of three phases, as described elsewhere [23]. In brief, *Phase 1* (level walking) involves level walking with increasing speed (3 min at 3.2 km/h and then accelerating at 0.33 km/h/min for the next 6 min), *Phase 2* (graded walking) consists of brisk walking (5.2–5.8 km/h) with increasing gradient (at a rate of 1.7% increased gradient/min for 6 min), and *Phase 3* (level running) involves level running with the treadmill speed increasing from 9 to 12.6 km/h for 4.5 min (average acceleration of 0.78 km/h/min). Transition between *Phase 2* and *3* entails a change in gradient of −10.2% over 30 seconds (to a level gradient), followed by a change in speed of +3.2 km/h over 30 seconds. The protocol finishes with a two minute standing recovery. The treadmill protocol is terminated early in the event that the participant wishes to stop or if 80% of the age-predicted maximal heart rate of the participant is exceeded. Energy cost (oxygen consumption) of this protocol is predicted from time (speed and incline) as reported elsewhere [23]. Cardiorespiratory fitness was estimated by extrapolation of the heart rate/oxygen consumption relationship to age-predicted maximal heart-rate. We only used data up to the time point which denotes the lowest common denominator between the baseline and follow-up visit for each participant.

Free-living physical activity was assessed at baseline and again at 12 weeks using combined sensing, following the pre-processing of the heart rate trace [29]. Data from the fitness test (see above) was used to individually calibrate heart-rate [23], and combined with acceleration in a branched equation framework [22] to estimate activity intensity (J/min/kg). Resulting time-series data are summarised as PAEE (kJ/kg/day) and time spent in sedentary (SED, in hours/day) and moderate-to-vigorous intensity physical activity (MVPA, in min/day), whilst minimising diurnal information bias caused by non-wear periods (segments of non-physiological data). Participants without individual calibration data have their free-living data processed using an age, sex, beta-blocker and sleeping heart rate adjusted group calibration equation for the translation of heart rate into activity intensity.

##### Anthropometric measures

Height was measured without shoes using a wall-mounted fixed rigid stadiometer. Weight and body fat percentage were measured using a bio-electrical impedance monitor without shoes and in light indoor clothing (Tanita BC-418MA). Waist circumference was estimated as the mean of two measurements taken with a D-loop measuring tape placed halfway between the lowest point of the rib cage and the anterior superior iliac crest with the participant in a standing posture and in light indoor clothing. If the two measurements varied by more than 3 cm a third measurement was taken and the mean of the three measures used.

##### Biological measures

Blood pressure and heart rate were calculated based on the mean of three measurements performed after 10 minutes of rest, with participants in a seated posture using an automatic sphygmomanometer (Omron) with the cuff placed on the dominant arm at the level of the heart. A resting electrocardiogram (ECG) was taken using a 12-lead ECG (Seca CT6i/CT6Pi).

Dietary intake of fruits and vegetables was assessed by plasma vitamin C levels. As humans are unable to synthesise vitamin C, and because the main source of vitamin C in the Westernised diet is fruits and vegetables [30], vitamin C levels provide an objective measure of dietary intake [31]. Plasma vitamin C was measured in venous blood collected into citrate tubes using a closed blood collection system (Monovette, Sarstedt, Germany). The plasma was then stabilised in a standardized volume of metaphosphoric acid and stored at −80°C. Plasma vitamin C concentration was measured using a fluorometric assay in monthly batches based on a method described by Vuilleumier & Keck [32] (PerkinElmer Victor 3 Plate reader).

HbA_1c_ was analysed in fresh blood by ion-exchange high-performance liquid chromatography (Tosoh G7 Haemoglobin Auto-analyser before August 2013 and Tosoh G8 Haemoglobin Auto-analyser after). Serum total-, LDL- and HDL-cholesterol and triglycerides were measured in fresh blood by enzymatic techniques (Dimension RxL Max Clinical Chemistry System (Siemens Healthcare Ltd) before December 2013 and Advia 2400 Chemsitry System (Siemens Healthcare Ltd) after). Serum fructosamine was analysed in monthly batches in serum stored at −80°C using an enzymatic method supplied by Randox, modified to run on the Siemens Dimension RXL. All biochemical analyses were conducted on venous blood collected using a closed blood collection system (Monovette, Sarstedt, Germany). Analysis of HbA_1c_ and lipids were conducted by the Department of Clinical Biochemistry, Addenbrooke’s Hospital, Cambridge; analysis of plasma vitamin C and serum fructosamine were conducted by the National Institute for Health Research Biomedical Research Centre’s Core Biochemistry Assay Laboratory, Cambridge. Both laboratories are fully accredited to perform biochemical analyses.

##### Demographic measures

Date of birth, sex, home living situation (i.e. living alone, with a partner or with friends), number of children aged <18 years living in the household, highest education level and ethnic origin were assessed by questionnaire.

##### Psychological measures

These include:

1. A TPB questionnaire, based on recommendations by Ajzen [33], to assess the theory’s major constructs as related to becoming more physically active: attitude, subjective norm, perceived behavioural control and intention. Each construct was assessed with five questions, measured on a 5-point Likert-type scale. Examples are: “Increasing how much physical activity I do over the next 12 weeks will be: harmful through to beneficial” (attitude); “The people whose opinions I value would approve of me increasing how much physical activity I do over the next 12 weeks”: strongly disagree through to strongly agree (subjective norm); “It is up to me whether or not I increase how much physical activity I do over the next 12 weeks”: strongly disagree through to strongly agree (perceived behavioural control); “I intend to increase how much physical activity I do over the next 12 weeks”: strongly disagree through to strongly agree (intention);
2. A similar TPB questionnaire designed to assess constructs related to physical activity self-monitoring. Each of the constructs (attitude, subjective norm, perceived behavioural control and intention) was assessed with questions measured on a 5-point Likert-type scale. Examples are: “Paying regular attention to how much physical activity I do over the next 12 weeks will be: harmful through to beneficial” (attitude); “The people whose opinions I value would approve of me paying regular attention to how much physical activity I do over the next 12 weeks”: strongly disagree through to strongly agree (subjective norm); “It is up to me whether or not I pay regular attention to how much physical activity I do over the next 12 weeks”: strongly disagree through to strongly agree (perceived behavioural control); “I intend to pay regular attention to how much physical activity I do over the next 12 weeks”: strongly disagree through to strongly agree (intention);
3. A conscientiousness questionnaire consisting of two questions measured on a 7 point Likert-type scale ranging from 1 (disagree strongly) to 7 (agree strongly). Questions were “I see myself as dependable, self-disciplined” and “I see myself as disorganised, careless”.

##### Self-reported health behaviours

Self-reported physical activity was assessed using the validated Recent Physical Activity Questionnaire (RPAQ) [34]. Functional status was assessed using the validated SF-8 health survey questionnaire [35] which includes items on general health, physical functioning, limitations due to physical health, bodily pain, vitality, social functioning, mental health and emotional stress. Perceived stress was assessed using the validated 4-item Perceived Stress Scale (PSS-4) [36]. The PSS-4 consists of four questions aimed at assessing feelings and thoughts during the past month, measured on a 5-point scale ranging from 0 (never) to 4 (very often). We used the Self-Report Habit Index (SRHI) [37] to assess habit strength for physical activity. The SRHI is a 12-item instrument that assesses prior behaviour, automaticity and identity expression using a 5-point scale ranging from 1 (strongly disagree) to 5 (strongly agree). Gym membership, smoking status, vitamin supplement use, frequency of self-weighing and current medication use were each assessed by questionnaire.

##### Degree of intervention use

Participants’ degree of intervention use will be assessed by: 1) degree of completion of the paper-based physical activity diary (for the Diary group); 2) total number of days in which physical activity was recorded on the accelerometer band from Imperative Health (Activity Band and Activity Band Plus groups), and 3) total number of times the Imperative Health website was visited (Activity Band and Activity Band Plus groups).

##### Other measures

Usual weekly leisure-time physical activity was assessed using the GLTEQ [24]. Presence of angina was assessed using the Rose Angina Questionnaire [38]. Participation in other research, GP advice not to exercise, beta-blocker use, pregnancy status, ability to walk briskly for ≥15 minutes, access to and ability to use a home PC and the internet, ability to use English language websites, likelihood of leaving job in next 4 months, self-report of doctor diagnosed diabetes, main study goal, job satisfaction, contact with other study participants, self-monitoring behaviour and use of physical activity advice websites were assessed by phone call/questionnaire.

##### Intervention evaluation

Following attendance at the follow-up visit, all participants were sent an evaluation questionnaire by email. Participants were asked four questions to determine satisfaction with participation in the study: ‘How much did you enjoy taking part in the study”, “How satisfied were you with the study”, “Would you like to continue with the study”, “Did taking part in the study raise your awareness of the amount of physical activity that you do?”. Participants in the three intervention groups completed additional randomised-group specific questions related to satisfaction and acceptability of the intervention.

#### Participant safety

The primary safety concerns for participants are cardiovascular and musculoskeletal events associated with the laboratory procedures of treadmill exercise testing and injuries sustained as a consequence of increasing physical activity during free-living (everyday life). The cardiorespiratory fitness test used was submaximal, and only undertaken following extensive screening procedures. If a participant had a positive Rose angina questionnaire [38] or an abnormal ECG then they were referred to a clinical member of the measurement team for a more detailed medical review. If there were clinical concerns then the treadmill test was terminated early and/or the participant referred to their GP. Supervising staff are trained and hold current cardio-pulmonary resuscitation certificates.

Given the nature of the interventions, the risk of excess injury is deemed to be minimal. Standard safety criteria set by Imperative Health (i.e., BMI ≤18 kg/m^2^ and high BP >160/100 mmHg) for use of their system were used as exclusion criteria at screening, thereby minimising any risk of harm to participants using these systems. Participants and their GPs were asked to inform the study team about any significant changes in health status over the 12 week course of the trial. Ranges for acceptable results were set for all clinical measures. If these ranges were exceeded the information was sent to the GP and the participant was informed and advised to consult their GP. The participant’s GP was informed of their patient’s involvement in the trial following the baseline visit if the participant had given written consent. The baseline and 12-week anthropometric and clinical measures were sent to the GP after the 12-week visit, unless the participant’s HbA_1c_ value was significantly raised at baseline (≥48 mmol/mol), in which case all available clinical and anthropometric values were sent to the GP after the baseline visit and the participant informed.

#### Sample size

Estimates used to calculate the total number of participants required for the *Get Moving* trial were taken from the ProActive trial [39], which had a similar study population to *Get Moving*. Participants’ mean (SD) PAEE at baseline in the ProActive trial was 0.116 (0.076) kJ/kg/min. 100 participants per group completing follow-up would allow detection of a difference of 0.03 kJ/kg/min in PAEE (which is equivalent to approximately 225–300 Kcals or 20 minutes of brisk walking per day) with 80% power at a 5% significance level. Since the analysis will adjust for baseline values in an ANCOVA model, and assuming a correlation between baseline and follow-up PAEE of 0.58 (as estimated in ProActive), 100 participants per group at follow-up would enable detection of a difference between groups in mean PAEE of 0.025 kJ/kg/min.

Participants’ mean (SD) for cardiovascular fitness at baseline in the ProActive trial was 3.2 (1.0) L/min with a correlation between baseline and follow-up of 0.88. After adjusting for baseline values, 100 participants per group enables detection of a difference between groups in mean cardiorespiratory fitness of 0.19 L/min with 80% power at a 5% significance level. Thus, we aimed to recruit 480 participants into the *Get Moving* trial with 120 in each of the four groups at baseline. This number will allow for an attrition rate of 17% (or 20 individuals) in each group, which we would expect given attrition rates in previous studies [27,39].

#### Statistical analysis

Baseline characteristics of the study population will be summarised separately within each randomised group. The co-primary outcomes, PAEE and cardiorespiratory fitness, will each be analysed using an ANCOVA that includes all participants in the group to which they were randomised, regardless of the intervention actually received (Intention-to-Treat analysis). The outcome in the ANCOVA model will be change (follow-up minus baseline) in PAEE (cardiorespiratory fitness), with the baseline value included as a covariate in the model. For each outcome a 3 degrees of freedom test will be performed of the null hypothesis that there is no difference between the 4 randomised groups. The ANCOVA model will also be used to derive estimates of the differences in mean change and 95% confidence intervals for each of the 6 pairwise comparisons: ‘*Diary*’ vs. ‘*Control*’, ‘*Activity Band*’ vs. ‘*Control*’, ‘*Activity Band Plus*’ vs. ‘*Control*’, ‘*Activity Band*’ vs. ‘*Diary*’, ‘*Activity Band Plus*’ vs. ‘*Diary*’ and ‘*Activity Band Plus*’ vs. ‘*Activity Band*’. Where baseline values of the outcome are missing, the missing indicator method will be used to enable these participants to be included in the analysis [40]. An analysis will be performed to check whether adjusting for age, sex and BMI (the randomisation stratifiers) in the ANCOVA model has any impact on the conclusions; if it has no impact, then they will not be included in the model.

For each continuous secondary outcome, the 6 pairwise differences between randomised groups will be estimated, together with 95% confidence intervals, using ANCOVA as described previously. Any continuous endpoints whose distribution is skewed will be log transformed prior to analysis, in which case a ratio of geometric means (and confidence interval) will be reported.

Subgroup analyses by sex and BMI (below/above median value) will be investigated for the 2 co-primary outcomes only.

Degree of use of the intervention will be summarised separately within each intervention group (i.e., the Diary, Activity Band and Activity Band Plus groups). The numbers and types of adverse events within each randomised group will be reported.

The primary analysis of outcomes will use an Intention-To-Treat population, which includes all participants in the group to which they were randomised, regardless of the intervention actually received. A secondary analysis of outcomes will use a Per-Protocol (PP) population. Inclusion in the PP population will be based on 1) degree of completion of the paper-based physical activity diary (for the Diary group); 2) total number of days in which physical activity was recorded on the accelerometer band from Imperative Health (Activity Band and Activity Band Plus groups), and 3) the total number of times the Imperative Health website was visited (Activity Band and Activity Band Plus groups). Degree of usage/completion will be defined once clean data are available (but before the start of any trial analyses), when the distributions can be inspected.

#### Data management and quality assurance

Each participant was assigned a unique numeric identifier code at the beginning of the *Get Moving* trial to enable link-anonymisation of data. All personal data is stored on an encrypted drive, and links to personal information are only available to the study co-ordination team. Consent forms and questionnaire data are stored in locked filing cabinets in secure Entacard-protected sites. All anthropometric and questionnaire measures are double-entered (with independent verification) by a quality-assured data entry company unaware of group allocation (Wyman Dillon Ltd, Bristol, UK). Random checks of entered data against the source document are performed, and the data are then assessed for outlying values together with appropriate range and consistency checks.

Trained personnel conduct the *Get Moving* trial according to standard operating procedures and the principles of Good Clinical Practice.

## Discussion

*Get Moving* will contribute to the evidence base on minimal contact interventions for physical activity by providing estimates of the effectiveness of three interventions: 1) A multi-component web-based intervention incorporating objective monitoring and graphical feedback of physical activity; 2) A version of the first intervention that consists only of objective monitoring plus web-based graphical feedback; and 3) Self-monitoring of physical activity using a paper diary. Comparisons between trial arms will enable us to address both pragmatic questions (how effective is each intervention compared with a no-intervention control condition?) and explanatory ones (do the additional components of the web-based intervention increase effectiveness over and above monitoring and feedback alone?; is objective monitoring and web-based feedback more effective than self-monitoring using a paper diary?). *Get Moving* will also provide information about potential mediation pathways.

Previous studies of minimal contact interventions have relied on self-report measures of physical activity. A major strength of *Get Moving* is the use of an objective measure of physical activity (PAEE measured using individually calibrated combined heart-rate and movement monitor data) and of cardiorespiratory fitness. Other strengths include the relatively large sample size and the use of robust methods of randomisation, allocation concealment and blinded outcome assessment.

The participants in *Get Moving* are physically inactive working adults recruited from a single large campus housing a teaching hospital, university departments and research institutes. However, the interventions are scalable and could be implemented in other occupational health settings and also in primary care.

## Abbreviations

ECG: Electrocardiogram
GLTEQ: Godin Leisure-Time Exercise Questionnaire
MVPA: Moderate-to-vigorous intensity physical activity
NHS: National Health Service
PAEE: Physical activity energy expenditure
PAM: Personal activity monitor
PA: Physical activity
RPAQ: Recent physical activity questionnaire
SED: Sedentary time
SF-8: Short Form 8 health survey questionnaire
SRHI: Self-Report Habit Index
TPB: Theory of planned behaviour
VO_2max.pred_: Predicted maximal cardio-respiratory fitness
WHO: World Health Organization
